# Positive perceptions of brown bears linked to long-term cohabitation in the Iberian Peninsula

**DOI:** 10.1038/s41598-025-16979-2

**Published:** 2025-10-02

**Authors:** Pedro Severino, Mariana Graça, Nuno Negrões, Tânia Barros, João Carvalho, Eduardo Ferreira, Roberto Hartasánchez, Bruno Malheiro, Luís Miguel Rosalino, Rita Tinoco Torres, Jenny Anne Glikman

**Affiliations:** 1 CESAM and Department of Biology, University of Aveiro, Campus Universitário de Santiago, Aveiro, 3810-193 Portugal; 2 Instituto de Ciências Sociais da Universidade de Lisboa (ICS-ULisboa), Av. Prof. Aníbal de Bettencourt, Lisboa, 1600-189 Portugal; 3 Fondo para la Protección de los Animales Salvajes (FAPAS), Ctra. AS-228, km.8,9, Santo Adriano Astúrias, Tuñón, 33115 Spain; 4 cE3c – Centre for Ecology, Evolution and Environmental Changes & CHANGE – Global Change and Sustainability Institute, Faculdade de Ciências, Universidade de Lisboa, Ed. C2, Campo Grande, Lisboa, 1749-016 Portugal; 5 Instituto de Estudios Sociales Avanzados (IESA-CSIC), Campo Santo de los Mártires 7, Cordoba, 1400 Spain

**Keywords:** *Ursus arctos*, Human-wildlife coexistence, Human-wildlife conflict, Expansion, Exposure, Attitudes, Psychology, Conservation biology

## Abstract

The brown bear (*Ursus arctos*) populations are expanding in the Iberian Peninsula, particularly in the Cantabrian Mountains. This ongoing expansion and the species’ return to Portugal could be jeopardized by a lack of sustainable coexistence strategies. To shed light on how experiences with bears have shaped human perceptions and attitudes toward this species, we distributed online questionnaires (*n* = 441) across Portugal and Spain. In addition, we evaluated differences in attitudes and perceptions among regions with varying levels of exposure to bears: those with (BAS) or without (NBAS) bear presence in Spain and those with (BAP) or without (NBAP) the potential to sustain bears in Portugal. Despite observing overall positive attitudes toward bears, Spanish respondents showed stronger attachment and receptiveness toward the species. Respondents from BAS, having more interactions with wild bears, were also more favourable toward sharing territory with them. Conversely, Portuguese respondents showed more neutral attitudes toward bear presence, as well as higher perception of risk and lower perception of benefit than previously reported. Our findings highlight the importance of understanding the social landscape in bears’ expansion range. As human-bear interactions increase, this knowledge is key to adopt proactive and targeted socio-ecological measures to address local community’s concerns.

**Supplementary Information:**

The online version contains supplementary material available at 10.1038/s41598-025-16979-2.

## Introduction

Many European large carnivore populations have persisted in human-shaped landscapes, despite enduring centuries of persecution due to their negative impacts on human livelihoods^1–3^. As a result of significant conservation efforts, some large carnivore populations have been experiencing an expansion in abundance and distribution^2,4,5^. In contexts of long-term cohabitation (i.e. sharing the same landscape) between humans and carnivores, local communities may tend to exhibit more positive attitudes and better acceptance of their presence^6–8^. One example of this pattern is the Apennine brown bear (*Ursus arctos marsicanus*) in central Italy, which have shared for centuries the Abruzzo, Lazio, and Molise National Park, and surrounding areas with local human communities and their activities^6^. In this region, studies show that residents like bears and strongly support their protection, especially in areas that received greater benefits to community livelihoods from bear presence, such as increased economic revenue from tourism^9^. Still, the recolonization of territories by large carnivores, where they have been absent for a long time, can trigger an escalation of conflicts due to increased interactions with communities that are neither accustomed nor adapted to sharing the landscape with these species^2,10^. For instance, Ivaşcu & Biro^11^ linked the historic use of endemic livestock guarding dogs to the tolerant attitudes towards large carnivores observed in the rural areas in the Romanian Carpathians. In fact, the loss or abandonment of traditional practices of livestock and agricultural protection has been correlated with a decrease in human tolerance toward large carnivores^12^, which could undermine people’s support toward the species and thus their conservation in the long-term^13^.

In the Iberian Peninsula, the range of brown bears (*Ursus arctos*) is divided into two small and historically isolated populations: the Cantabrian population (NW Spain) and the Pyrenees population (NE Spain and SW France). Both populations have faced significant threats from hunting pressure and habitat fragmentation^14^. Particularly, the Cantabrian brown bear population, once on the brink of extinction in the 20th century, is now undergoing a natural expansion^14–16^. Since the mid-1990s, a positive shift in public opinion has contributed to the recovery of the western and eastern subpopulations^14^, reflected in a continuous increase in the number of females with cubs of the year^17,18^, an expansion on their spatial distribution^19^, and the reestablishment of the bidirectional gene flow between subpopulations^15,20^. This recovery, however, has been more pronounced for the western nucleus^15,17^. Díaz-Fernández et al.^19^ showed that most of the current and predicted future dispersal is centred around individuals, primarily males, from this subpopulation expanding southwest. Until recently, the last confirmed brown bear observation in Portugal dated back to 1843 and involved a male believed to have dispersed from Spain^14,21^. However, in 2019, almost 200 years later, a dispersing male from the Cantabrian western nucleus was observed in northwestern Portugal (Montesinho Natural Park)^22^. Currently, the frequent observations of individuals from this subpopulation near the Portuguese-Spanish border^23^ suggest a growing potential for more frequent future incursions into Portugal.

Local communities’ attitudes toward brown bears have been extensively monitored across Europe^7,13^. While some studies described a majority of positive attitudes, such as in Italy^6,24^, Romania^8^, and Croatia^25^, others reported a higher prevalence of negative attitudes, as seen in Norway^26^ and in Sweden^27^. While variance of attitudes toward bears tends to be greater at large spatial scales, i.e. countries^28^, , this heterogeneity is also apparent at a regional scale. Within the Iberian Peninsula, the generally well-regarded and welcomed Cantabrian bear population^23^ evokes opposite reactions to the frowned upon Pyrenees bear population^29,30^. Within the French Pyrenees, Piédallu et al.^29^ found that individuals residing in counties with continuous bear presence had more positive attitudes toward bears compared to those in neighbouring counties where bears had been reintroduced more recently. To the best of our knowledge, only one study conducted in Northern Portugal has examined some actors’ perception and attitudes toward bears following the confirmed incursion into Portugal^22^. This study reported overwhelmingly positive attitudes and strong consensus among the surveyed actors, primarily academic researchers, NGO members, and national administrators. This finding somehow contrasts with most existing evidence on human-bear relationships in Europe, which indicates more negative perceptions evaluation of the species in areas undergoing recolonizing^7,10^. As local communities adapt to sharing the landscape with returning carnivores, their attitudes are likely to change^31^. Therefore, it is vital to further explore the willingness of local people to accept bear presence and the factors influencing these perceptions in this expansion region. A broader understanding of social acceptance of the species will help guide transboundary conflict prevention and tailored mitigation efforts in areas at higher risk of conflict, before strong negative beliefs cement, and thus promoting coexistence with this carnivore^10,31^.

Our study aimed to assess whether varying levels of exposure — defined as the frequency of an individual’s interactions with a species^32^ — to brown bears influence perceptions and attitudes toward the species and its presence in the Iberian Peninsula. We identified four regions for questionnaire administration: two in Spain (one with bear presence and one without) and two in Portugal (one with and one without the potential to sustain a bear population in the future). Focusing on the ongoing expansion of the western subpopulation of the Cantabrian bear and its dispersal into Portugal, we characterized and compared residents’ experiences with bears in each region. We then performed statistical analyses to determine whether: (i) perceptions and attitudes toward bears differ based on country of residence, and (ii) perceptions and attitudes toward bears vary between regions with different levels of exposure to brown bear within each country. We expect that Spanish residents will exhibit more positive attitudes and perceptions toward bears than Portuguese residents. Additionally, we anticipate that residents from the Spanish region with bear occurrence (BAS) will express the most positive attitudes and perceptions among all the study areas, consistent with previous research on human attitudes in regions with persistent bear populations^6–8^. Conversely, we predict that residents in the Portuguese region where bears may potentially appear in the future (BAP) will exhibit more negative attitudes and perceptions than other Portuguese residents, as they would be the ones who would face the potential risks and costs associated with bear presence^29,33^.

## Methods

### Study area

**Fig. 1:**
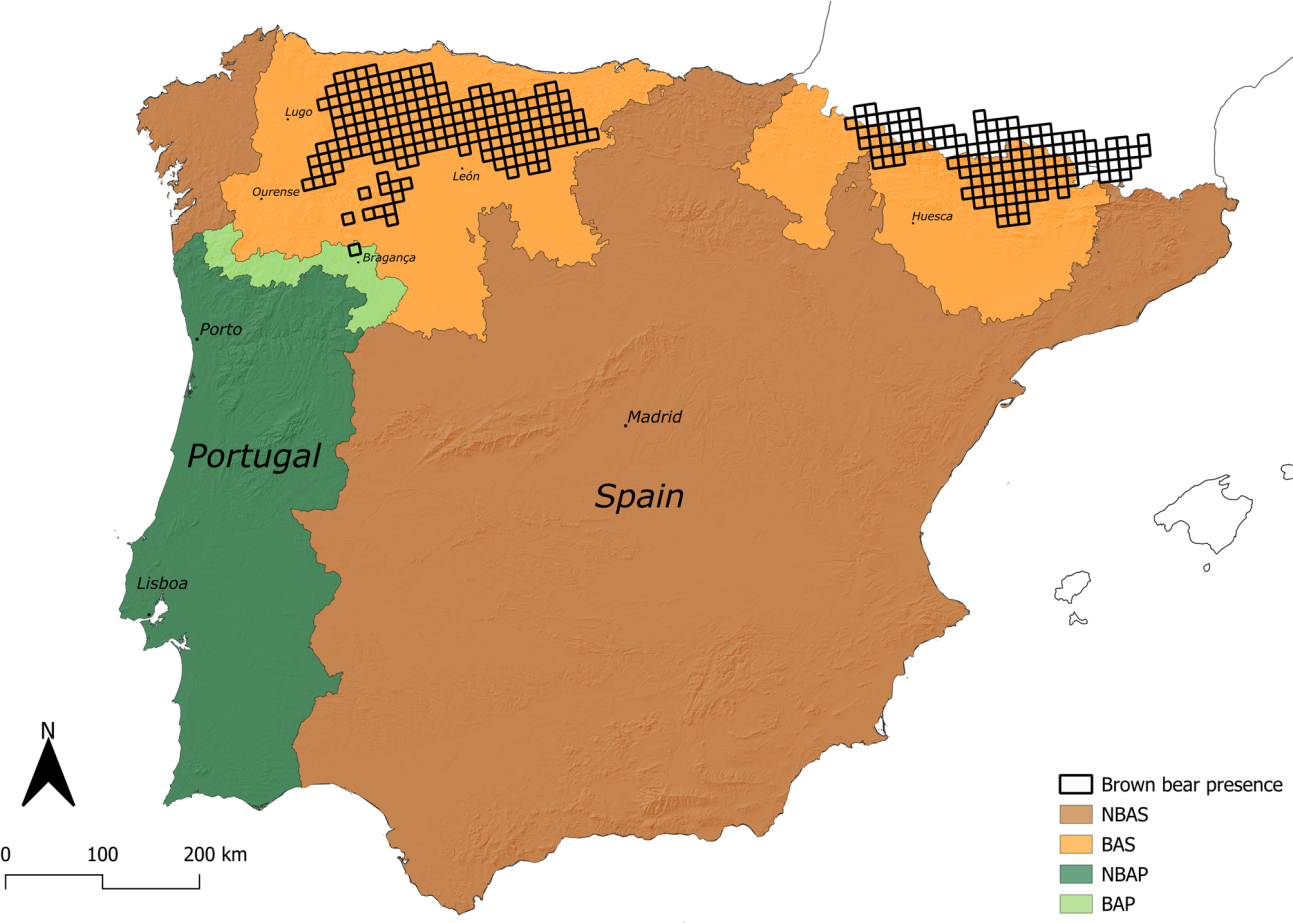
Map of the study area in the Iberian Peninsula and its subdivision into the four sampling regions: BAS = Area with bear occurrences in Spain; NBAS = Area without bear occurrences in Spain; BAP = Area with high potential for bear occurrence in Portugal; NBAP = Area with null or very low potential for bear occurrence in Portugal. The black-outlined grid represents the brown bear distribution range of both the Cantabrian (left) and the Pyrenees (right) populations. The map was created in QGIS Desktop 3.40.9 (https://qgis.org/) using the Digital Elevation Model published by USGS (https://portal.opentopography.org/raster?opentopoID=OTSRTM.082015.4326.1) with a 1-arc/second resolution (± 30 m). Bear presence data was retrieved from Kaczensky et al.^5^.

The study area includes the Iberian Peninsula, encompassing four distinct regions defined based on the levels of human exposure to brown bears (Fig. 1). In Spain, we utilized the most updated datasets on brown bear presence, which compile records of direct observations, damages, and indirect signs of presence^19,34^, to differentiate between provinces where bear presence is confirmed (BAS) and those without bears (NBAS). In Portugal, we identified municipalities where brown bears could potentially occur (BAP) based on three main factors: proximity to the Cantabrian bear population range^22,23^, the dominance of habitat types similar to the ones found in the bear range^35,36^, and the low human density^37^. This region includes the area where, in 2019, a male bear incursion occurred and is therefore expected to experience increased exposure to bears in Portugal in the future. The remaining Portuguese territory (NBAP) was deemed unlikely to support future bear expansion.

### Theoretical framework

We primarily used the Psychological Framework of Carter et al.^38^ and the Wildlife Tolerance Model of Kasky et al.^33^ to identify the key variables for design of our questionnaire and as guidance to explore the potential relationship amount those variables. Both empirical frameworks established a relationship between past experiences and individual tolerance toward a species, a type of human-wildlife relationship that fosters coexistence^39^. However, due to the inconsistent definition of tolerance in the literature^39,40^, we chose instead to measure people’s attitudes toward the presence of brown bears, as this can be interpreted as a precursor to tolerance or acceptance behaviour^40^.

Recent literature on human-carnivore interactions has shifted its focus away from the negative impacts of wildlife on humans and vice versa^41^ to the positive impacts of human-carnivore relationship toward coexistence^39,41,42^. Despite its validity, the classical framing of the interactions between wild animals and humans as antagonistic, implied in both conceptual models, may negatively affect peoples’ psyche and influence their risk perception of these species^43^. Therefore, in this study, we used experiences of bear sightings and their perceived impact as a measure of exposure to the species, aiming to establish a link between exposure to bears and individuals’ beliefs, perceptions, and attitudes toward the animals and their presence. Since wild bear sighting is uncommon, more so for people that live outside of the bear’s range or in another country (i.e. Portugal), many individuals may associate their experience with bears with seeing one in the zoo or another facility where the animals are in captivity. To account for this, we asked respondents to differentiate between the two types of encounters, as they are often interpreted differently^44^.

Attitudes are defined as a positive or negative evaluation of a subject^45^. These evaluations can be broken down and assessed indirectly through two components: the affective component, i.e. the emotional response to bears; and the cognitive component, usually referred to as beliefs^31^ i.e. the factual or perceived factual information available to a person about bears^46^. We analysed these components of the attitudes toward bears separately, as they explain different factors that influence human behaviour^47,48^. Additionally, while the perception of benefits and risks could be considered part of an individual’s cognition regarding the attitude object^49^, we also analysed these perceptions separately, as several studies have demonstrated that they help explain most patterns of individual tolerance/acceptance of carnivores^33,38,42^.

### Questionnaire structure

The questionnaire consisted of 44 closed and open-ended questions divided into four sections: a) experiences with bears; (b) knowledge about the species; (c) perception of economic benefits/risks and attitudes toward bears and their presence; and (d) sociodemographic characterization of respondents (Supplementary Data S1). For the aim of this study, we analysed the variables related to the experiences, perceptions and attitudes toward bears and their presence, as described in the theoretical framework section.

Participants were asked whether they had ever seen a bear in the wild and in captivity (yes = 1, no = 0) and, through a multiple-choice question, how frequently. Furthermore, we measured the effect of those experiences using a 5-point Likert-type scale, spanning from 1 = strongly negative to 5 = strongly positive. Respondents’ relationships with nature were also assessed using a multiple-choice question about the frequency of nature contact, as this may influence attitudes toward species such as bears^50^. The affective component of attitudes toward bears was assessed by asking the respondents about their feelings toward the species, while the cognitive component was measured through two belief statements regarding the inherent rights of bears to exist and the importance of their conservation. We evaluated individual attitudes toward bear presence using two attitudinal items: one regarding bear presence in the respondent’s country and another concerning their presence in the respondent’s municipality. We asked respondents whether they perceived any economic benefits and/or risks associated with sharing the region with the species. All these questions were measured using a 5-point Likert-type scale ranging from 1 = strongly disagree/negative to 5 = strongly agree/positive. Additionally, to detect a potential “Not In My Backyard” (NIMBY) effect within the areas, i.e. whether individual’s acceptance of bears is contingent on their presence only if they do not occur in the vicinity of their residence, we asked participants, though a multiple-choice question, to identify the minimum distance from their area of residence at which bear presence would become a cause for concern. This effect has been observed in relation to other large carnivore species in the Iberian Peninsula^51^. Additionally, we also performed a qualitative analysis of bear influence on local culture by asking respondents to recall myths, legends or histories from their childhood. As evidenced for other large carnivore species e.g.^52^, the cultural identity of the species could influence people’s attitudes and therefore allow us to enhance the quantitative analysis of differences, particularly between countries.

### Data collection and analysis

Between April and September 2021, we made two versions of the same online questionnaire available, one in Portuguese and the other in Spanish, using the LimeSurvey platform, which ensures data protection and anonymity to the respondents through encryption. The questionnaire was reviewed and approved by both the Institutional Review Board of Miami University, Ohio (protocol number 03848e), and the Ethics Committee of the University of Aveiro. All methods were performed in accordance with the relevant guidelines and regulations, namely the ethical principles of voluntariness and confidentiality, with all respondents providing informed consent prior to participation. To enable a robust statistical test of our hypotheses across such a large study area, we initially planned to conduct our survey using online questionnaires and compare these findings with in-person interviews to detect possible biases associated with the online method^51^. However, because most of our study’s sampling period overlapped with a mandatory travel ban and isolation period due to the COVID-19 pandemic, we were unable to conduct the in-person interviews. For the same reason, we did not perform actors’ comparison, as we could not ensure representativeness of all key interest groups across the study areas.

Participation was restricted to the adults (≥ 18 years) residing in our study area. Respondents were asked to submit their responses only once to minimize unintentional fraudulent results^53^. We used two different methods of dissemination of the questionnaire to increase the representativeness of the general public in each region. First, we distributed the survey link via the mailing lists of several institutions (e.g., universities, hunting associations, agricultural cooperatives, NGOs, local governments, and wildlife management organizations) that operate locally in the four regions and nationally in each country. Additionally, we shared the same link on the social media platform Facebook, in various pages and groups that discuss topics related to bears and wildlife (e.g., hunting, livestock, honey production, and wildlife enthusiasts).

Likert-type scales were treated as quantitative ordinal variables and converted to a new scale ranging from − 2 to 2^54^. For single-item variables, including experiences, perceived benefits and risks, feelings toward bears, and the minimal accepted distance from home, we used a Chi-square independent test to assess differences between countries. We used Cramer’s V to quantify effect sizes, with thresholds of V ≥ 0.1 for minimal, V ≥ 0.15 for typical, and V ≥ 0.25 for substantial effects^55^. In the absence of significant differences between countries, we evaluated the differences between the four regions using Kruskal-Wallis tests, interpreting effect sizes using ETA (η), where η ≥ 0.1 was considered minimal, η ≥ 0.24 typical, and η ≥ 0.37 substantial^54^. For the cognitive component of attitudes toward bears and attitudes toward bear presence, which were both measured with multiple items, we used the independent samples t-test to compare average responses between countries, with Cohen’s d effect sizes to quantify the magnitude of these differences (d ≤ 0.2 as minimal, d ≤ 0.5 as typical, and d ≥ 0.8 as substantial^54^. Similar to the single-item variables, when no significant differences were found between countries, we conducted one-way ANOVAs to investigate differences among the four regions, with the calculus of the ETA effect size. We performed Games-Howell post-hoc tests to identify significant pairwise differences among regions for all variables, assuming unequal variance between samples. All statistical analyses were conducted using IBM SPSS, Version 28.0 (IBM Corp., Armonk, NY, USA), with a significance level of *p* < 0.05.

## Results

From a total of 657 submitted questionnaires (*n* = 372 in Portuguese and *n* = 285 in Spanish), we selected only those that were fully completed, resulting in a total of 441 questionnaires (*n* = 252 and *n* = 189, respectively). These were distributed across the four regions as follows: BAS = 65; NBAS = 124; BAP = 54; NBAP = 198.

The demographic characteristics of the respondents are presented in Table 1. Across most regions, the average respondent age ranged between 45 and 55 years old, except for the NBAP area, which had a significantly younger average of 35 years. Gender representation varied by country: Spanish regions, especially NBAS, had a higher proportion of male respondents, whereas Portuguese regions, particularly NBAP, showed a female majority. Overall, the majority of respondents (68%) held university degrees, with the NBAS and NBAP areas exhibiting the highest levels of educational attainment. The association between demographic characteristics and attitudes and perceptions toward bears is detailed in Supplementary Table S1.

**Table 1:** Demographic characteristics of respondents from the four regions: BAS = area with bear occurrences in spain; NBAS = area without bear occurrences in spain; BAP = area with high potential for bear occurrence in portugal; NBAP= area with null or very low potential for bear occurrence in Portugal.

*N*	BAS 65	NBAS 124	BAP 54	NBAP 198
Gender (Male)	69%	73%	50%	39%
Age	50 years (SD = 10)	48 years (SD = 12)	47 years (SD = 13)	35 years (SD = 14)
Education (University degree)	51%	68%	57%	77%

Our results also showed that stories involving bears were absent from the majority of respondent’s childhoods. Nevertheless, a higher proportion of Spanish residents recalled legends or myths about bears (33%; *n* = 63) than Portuguese residents (18%; *n* = 45). A common reference among residents of both countries was popular children’s stories (*n* = 20 and *n* = 17 in Spain and Portugal, respectively), such as “Goldilocks and the Three Bears” or “The Jungle Book”, which portrayed bears as kind and caring animals. In Spain, some residents (*n* = 5) recalled the Asturian myth of the death of King Favila, in which his death was supposedly caused by a confrontation with a bear during a hunting trip. In the BAS area, stories about bears stood out from other regions, as they featured one or more individual animals known to the local community, sometimes even associated with a name (*n* = 7):

*“There were always talks about Paca and Tola*,* the two Asturian orphan bears.” (respondent 129ES*,* woman*,* 36 years old)*.

“There was a bear «crouched» near my village. If you passed by it, it would do you no harm… Sometimes, it would go into the corn fields to eat the corn.” *(respondent 9ES*,* male*,* 45 years old)*.

Contrarily, while stories focused on the dangers of bears were present among Spanish residents (*n* = 6), they were more prevalent among the Portuguese residents. More than half of the Portuguese respondents who recalled stories about bears mentioned that bears would attack or behave aggressively toward people (*n* = 10) or that bears were remembered for attacking beehives (*n* = 14), leaving a lasting mark on the cultural memory of their villages:

“The bears were horrible creatures. They ate humans…” *(respondent 107PT*,* woman*,* 54 years old)*.

“That the bears showed up at night to devour people that behaved badly” *(respondent 215PT*,* female*,* 52 years old)*.

“They existed, because in the region where I live there are some small land plots with high walls where there used to be beehives. They would call them «sila dos ursos» that were there to prevent that the bears reached the beehives and ate the honey” (*respondent 330PT*,* male*,* 30 years old)*.

### Experience with bears

Almost all respondents (83%; *n* = 365) had seen a bear in captivity at least once, with the majority having seen bears between 2 and 5 times (58%; $\:\stackrel{-}{x}$ = 2.33). A significant disparity was observed between individuals from Portugal and Spain, as a lower proportion of Portuguese respondents (76%) had seen a bear in captivity compared to Spanish respondents (92%; χ^2^ [1, *n* = 441] = 20.04; *p* < 0.001; V = 0.21). Furthermore, within the Portuguese population, fewer residents from the BAP area (59%) reported this experience compared to those from the NBAP area (80%; *p* = 0.002).

Observing bears in the wild was less common, with only 17% (*n* = 75) of respondents stating to have had such an experience. Nonetheless, we found that it was significantly more frequent among Spanish respondents (31%) than among Portuguese respondents (6%; χ^2^ [1, *n* = 441] = 47.32; *p* < 0.001; V = 0.33). Additionally, residents of the BAS area were more likely to have seen a bear in the wild (42%) compared to those in the NBAS area (26%; *p* = 0.03).

**Fig. 2:**
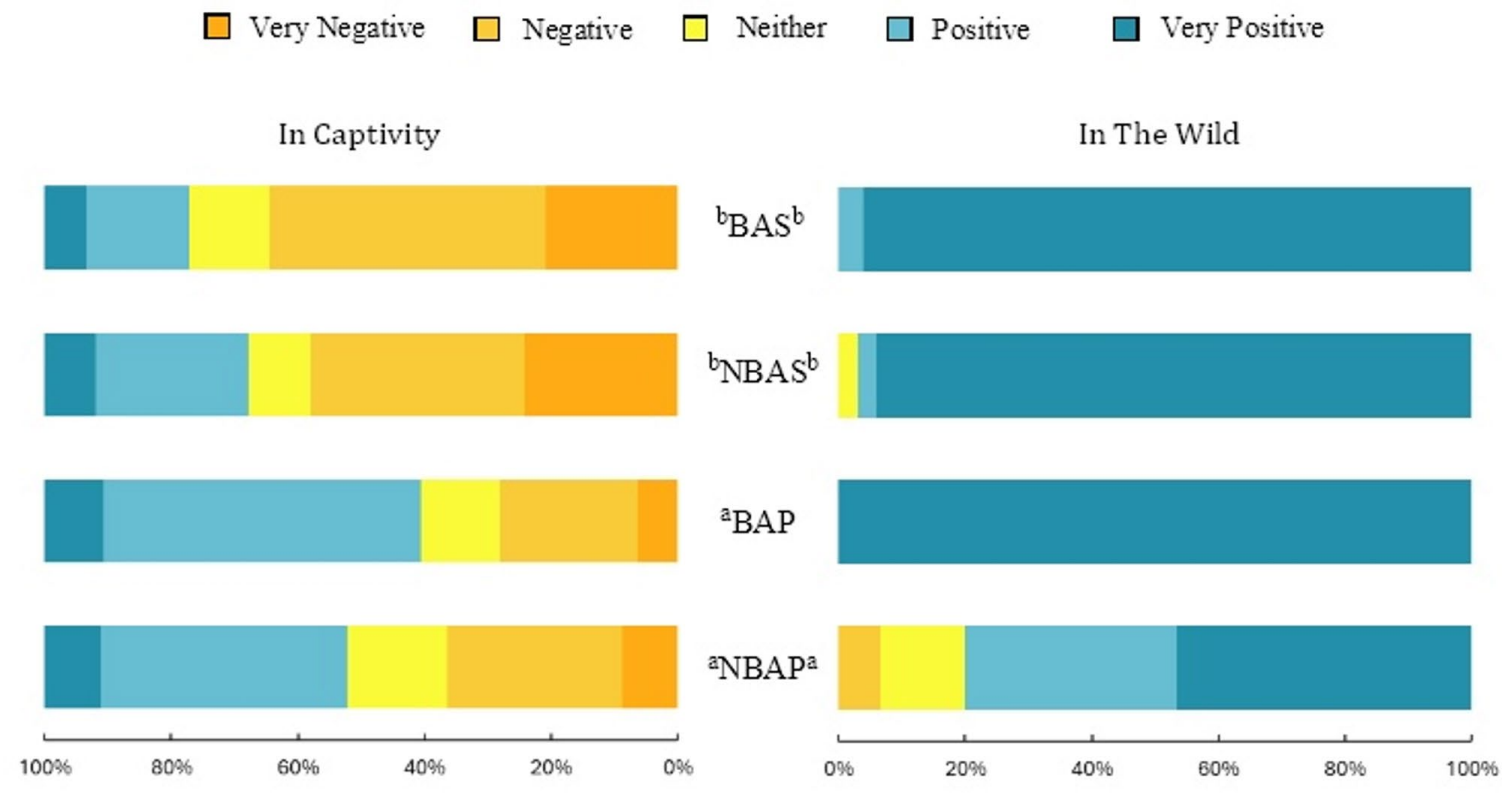
Differences of the perceived impact of respondents’ encounters with bears in captivity (left) and in the wild (right) according to the region of residence: BAS = Area with bear occurrences in Spain; NBAS = Area without bear occurrences in Spain; BAP = Area with high potential for bear occurrence in Portugal; NBAP = Area with null or very low potential for bear occurrence in Portugal. Regions with different letter subscripts are significantly different at *p* = 0.05, based on the Games-Howell post-hoc test.

The perceived meaningfulness of bears encounters varied depending on the context of the experience (Fig. 2). Nearly half (47%, *n* = 365) of the respondents classified seeing a bear in captivity to be a “negative” or a “very negative” experience. In contrast, the vast majority (90%, *n* = 75) of those who had seen a bear in the wild rated the experience as “positive” or as “very positive”. Significant differences between countries were detected: Spanish respondents classified the experience of seeing a bear in the wild more positively and seeing one in captivity more negatively than the Portuguese participants (*p* < 0.001; V = 0.28 and V = 0.78, respectively). The different opinions on the impact of seeing a bear in the wild were less evident when comparing the four regions, likely due to the low number of people who had such experience. However, the NBAP region contributed the most to the observed differences between the countries (Fig. 2).

When analysing the respondent’s frequency of contact with nature, we found that a higher proportion Spanish respondents (77%) reported spending time in nature at least every week compared to Portuguese participants (64%). However, the effect size of these differences was below the threshold form minimal practical significance (χ^2^ [3, *n* = 441] = 36.51; *p* < 0.001; V = 0.08), indicating that the detected differences were not meaningful. Moreover, the pairwise analyses detected significant differences between all regions (*p* < 0.001). Still, more than half of the participants from both countries (69%; *n* = 306) spent time in nature on a weekly basis. Particularly, BAP residents were in majority spending time in nature every day (63%).

### Affective and cognitive components of attitudes toward bears

**Fig. 3:**
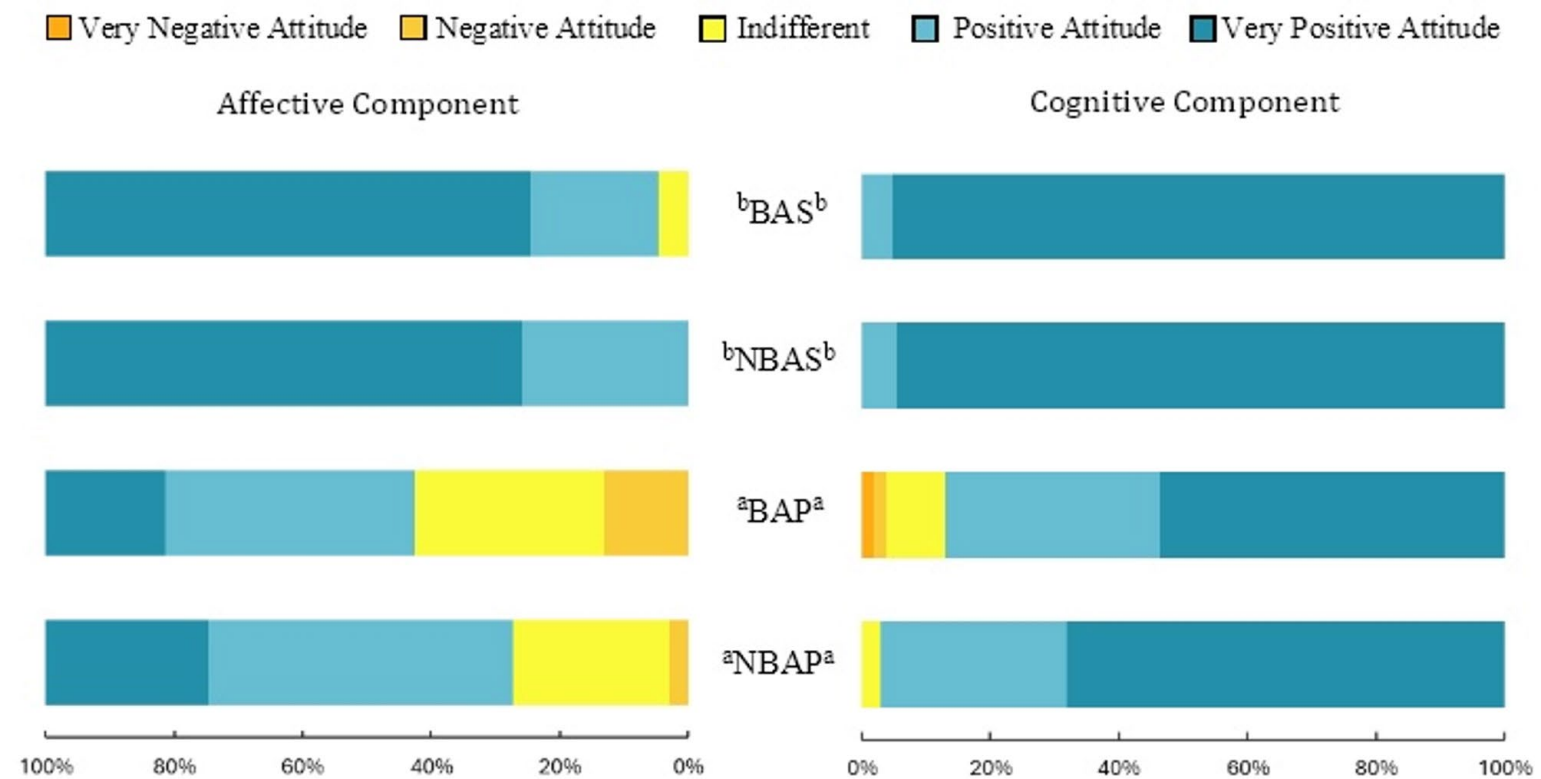
Differences in the affective (right) and cognitive (left) components of people’s attitudes toward bears between the four regions: BAS = Area with bear occurrences in Spain; NBAS = Area without bear occurrences in Spain; BAP = Area with high potential for bear occurrence in Portugal; NBAP = Area with null or very low potential for bear occurrence in Portugal. Regions with different letter subscripts are significantly different at *p* = 0.05, based on the Games-Howell post-hoc test.

Overall, most respondents shared positive feelings about the species (82%; Fig. 3), but having greater affection for bears was more predominant among Spanish people ($\:\stackrel{-}{x}$ = 1.73; SD = 0.48) compared to Portuguese participants ($\:\stackrel{-}{x}$ = 0.88; SD = 0.83; χ^2^ [3, *n* = 441] = 125.36; *p* < 0.001; V = 0.53). A pairwise comparison between regions revealed the same pattern (χ^2^ [3, *n* = 441] = 128.27; *p* < 0.001; η = 0.53), with no significant differences observed between regions within the same country.

A similar pattern emerged regarding beliefs about the right of bears to exist in the country and their conservation (Fig. 3), with substantial differences in the cognitive index score between countries (t [341] = -10.65; *p* < 0.001; d = 0.95). Spanish residents were almost unanimously in agreement with these beliefs, resulting in a very high average score ($\:\stackrel{-}{x}$ = 1.93; SD = 0.05). In contrast, Portuguese residents were significantly less in agreement with these beliefs ($\:\stackrel{-}{x}$ = 1.47; SD = 0.39). No statistical differences were found between regions within the same country.

### Benefit and risk perception

**Fig. 4:**
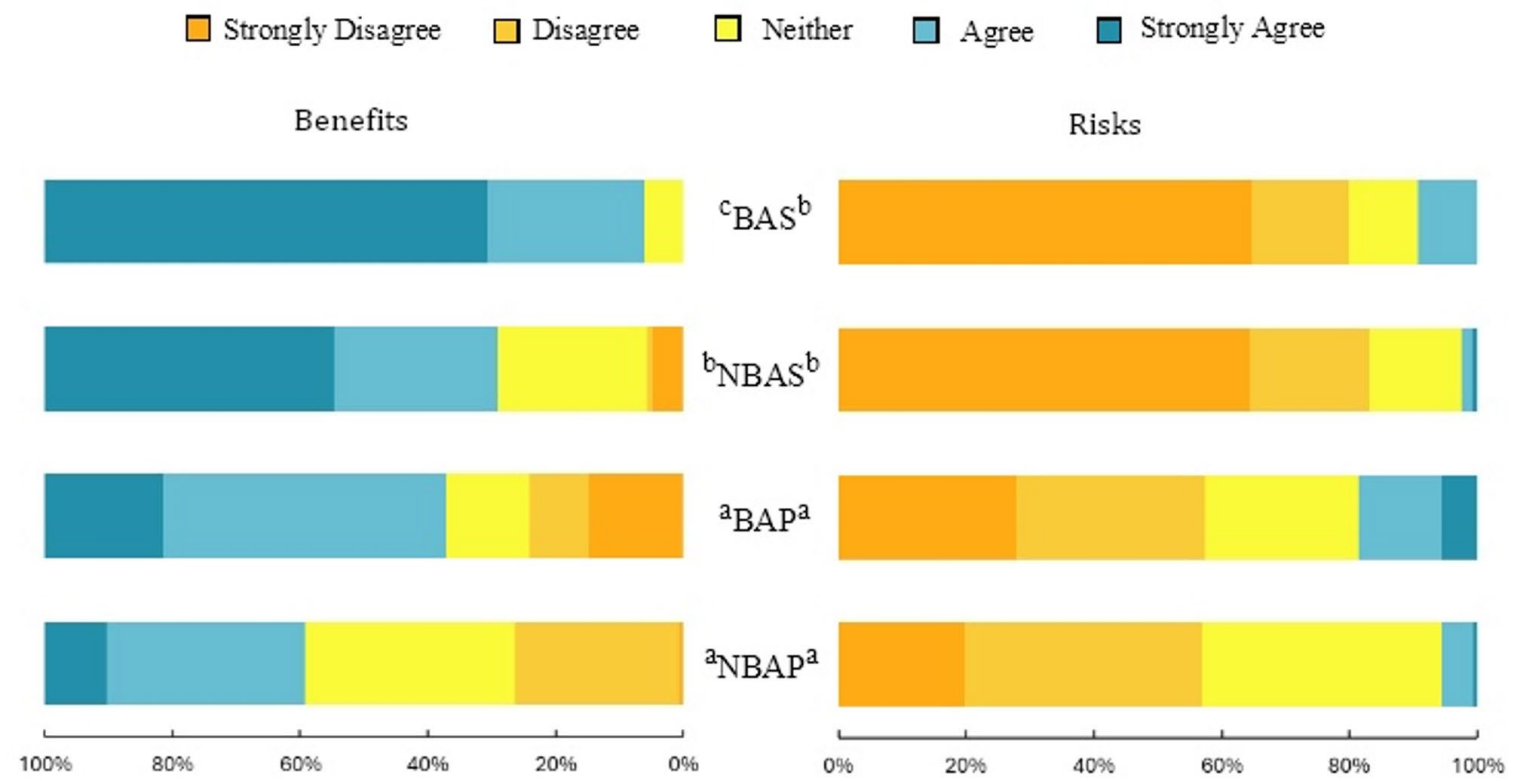
Differences of the perception of benefits (right) and risks (left) among people from the four regions: BAS = Area with bear occurrences in Spain; NBAS = Area without bear occurrences in Spain; BAP = Area with high potential for bear occurrence in Portugal; NBAP = Area with null or very low potential for bear occurrence in Portugal. Regions with different letter subscripts are significantly different at *p* = 0.05, based on the Games-Howell post-hoc test.

Overall, the perception of benefits outweighed the perception of risks in both countries (Fig. 4), but this was more pronounced among Spanish respondents, who were mostly in agreement with the existence of benefits ($\:\stackrel{-}{x}$ = 1.25; SD = 0.98) and disagreed with the existence of risks ($\:\stackrel{-}{x}$ = 1.41; SD = 0.91). Portuguese respondents were more divided in their opinions, having an overall more neutral position regarding both potential benefits ($\:\stackrel{-}{x}$ = 0.15; SD = 1.15) and risks ($\:\stackrel{-}{x}$ = 0.68; SD = 0.94) compared to Spanish counterparts. The strength of the observed differences between countries was substantial for both perceived benefits and risks (χ^2^ [4, *n* = 441] = 114.25; *p* < 0.001; V = 0.33 and χ^2^ [4, *n* = 441] = 84.78; *p* < 0.001; V = 0.44, respectively). Furthermore, residents of the BAS area were more likely to agree that the bear presence brings benefits to the region ($\:\stackrel{-}{x}$ = 1.63; SD = 0.60) compared to NBAS residents ($\:\stackrel{-}{x}$ = 1.06; SD = 1.07).

### Attitudes toward bear presence

**Fig. 5:**
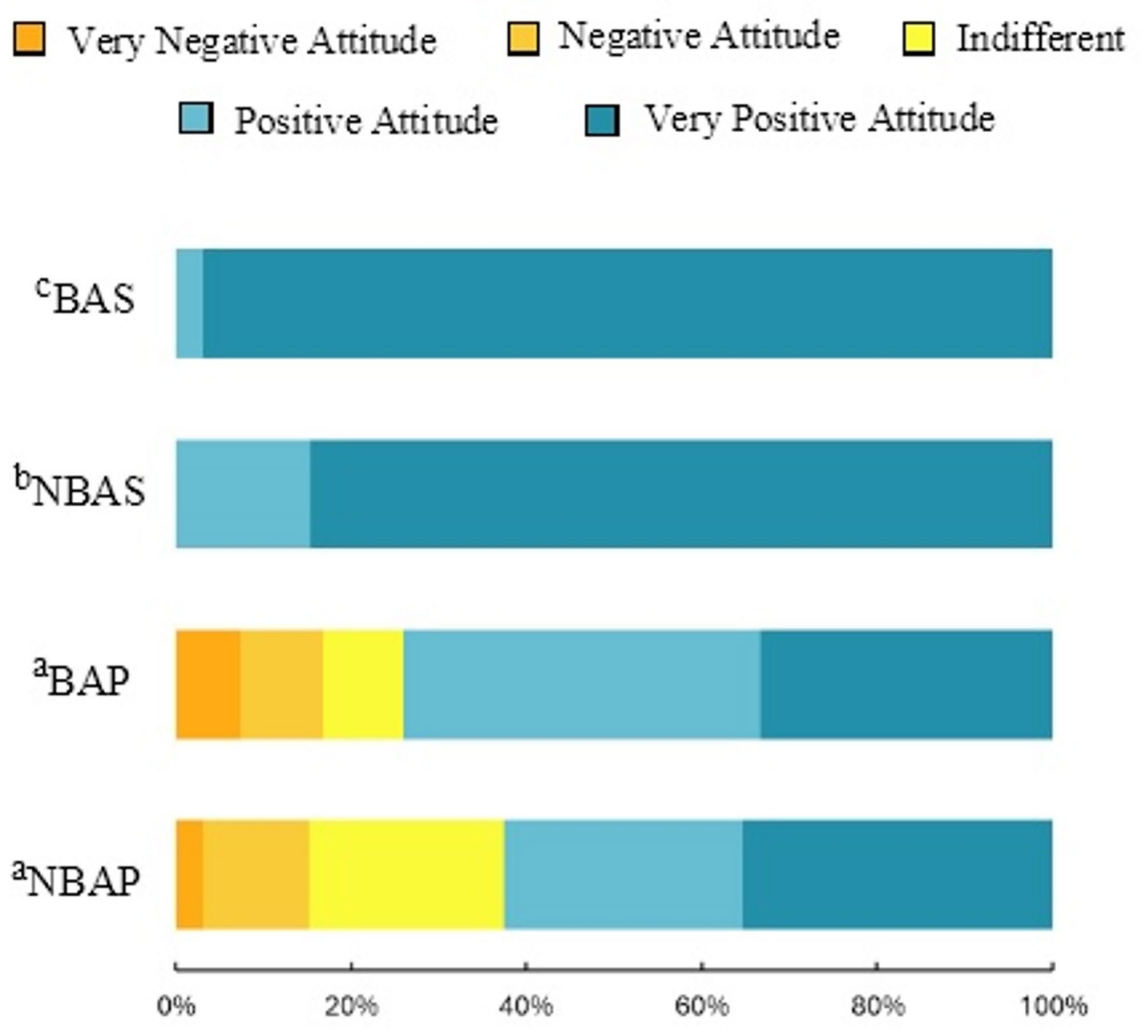
Differences in people’s attitudes toward bear presence between the four regions. BAS = Area with bear occurrences in Spain; NBAS = Area without bear occurrences in Spain; BAP = Area with high potential for bear occurrence in Portugal; NBAP = Area with null or very low potential for bear occurrence in Portugal. Regions with different letter subscripts are significantly different at *p* = 0.05, based on the Games-Howell post-hoc test.

A majority of respondents expressed positive attitudes toward brown bear presence (Fig. 5). However, a significant difference emerged between countries: Portuguese respondents displayed notably more neutral attitudes regarding bear presence ($\:\stackrel{-}{x}$ = 0.75; SD = 1.03), compared to the strongly positive attitudes among Spanish respondents ($\:\stackrel{-}{x}$= 1.85; SD = 0.36; t [328] = -15.79; *p* < 0.001; d = 1.36). Furthermore, within the Spanish population, attitude intensities varied significantly between regions. Respondents from the BAS area exhibited stronger positive attitudes ($\:\stackrel{-}{x}$ = 1.95; SD = 0.20) than those from the NBAS area $\:\stackrel{-}{x}$( = 1.80; SD = 0.42; *p* < 0.01).

A similar pattern was observed regarding concerns about bear proximity to peoples’ residential areas. On average, Spanish respondents indicated a minimum distance of 9.73 km (SD = 29.48) before experiencing worry over bear proximity. Within Spain, BAS respondents established a significantly closer threshold ($\:\stackrel{-}{x}$= 4.95 km; SD = 17.10) compared to NBAS respondents ($\:\stackrel{-}{x}$= 12.23 km; SD = 33.85; *p* = 0.01). In contrast, Portuguese respondents demonstrated a significantly more stringent threshold ($\:\stackrel{-}{x}$ = 29.17 km; SD = 80.66), evidencing a greater discomfort with bear proximity compared to their Spanish counterparts.

## Discussion

Overall, public opinion on brown bears and their presence throughout the Iberian Peninsula was positive. This is aligned with other studies in Southern Europe^6,8,24,25^ showing that brown bears are generally viewed through a positive lens. Even so, our results highlight the importance of continuous exposure to this species in fostering local support that can be harnessed toward coexistence. Our first hypothesis was supported, since Spanish respondents, who had more frequent and direct experience of seeing bears in the wild, expressed stronger positive beliefs, perceptions, and attitudes than Portuguese respondents. A similar pattern was observed between Spanish regions with and without bear occurrence. However, in Portugal, contrary to our initial prediction, no significant difference of any measured variable was detected between residents of regions with and without the potential to sustain bears in the future. Nevertheless, these results still support our second hypothesis, as no significant difference in exposure was observed between the two Portuguese regions. This suggests that the distinction we made between areas in Portugal based on socio-ecological factors may not yet be relevant for the local populations, as the return of bears to Portugal is not widely perceived as a reality. When comparing the time respondents spend in nature, no meaningful differences were observed between countries, as a high proportion of respondents reported weekly contact with nature. This limits the ability to observe a potential link between these differences to variations in attitudes and perceptions toward bears that were reported previously with this species^50^. Nevertheless, relationships with nature, shaped not only by quantity, but also the quality of time spent in nature, can contribute to the development of positive environmental attitudes^56^. A more representative sample, including both rural and urban residents, who often differ in their perceived value of connectedness to nature^56^, could provide stronger evidence for this correlation.

### Positive experiences mediate attitudes

Our results indicate that the more favourable attitudes occur in regions where people’s perception of the interactions with brown bears were more positive and where encounters are more frequent, particularly the BAS area, concurring with the findings by Hull et al.^57^. Moreover, respondents who rated seeing bears in the wild as very positive experience were the same individuals who held the most negative views about seeing them in captivity. This suggests a strong attachment to the wilderness and potential concern to bears well-being. The preference for seeing bears in the areas where they naturally occur in Spain, may stem from the perception that enclosed animals are often connected to be in poor health^44^.

In some regions in Spain, humans have historically cohabited with bears. This long-standing sympatry can foster traditional empirical knowledge about the species, its behaviour, and how to adapt to their presence^8,11,12^. Moreover, persistent interactions with bears can also evoke and enhance relational values - “preferences, principles, and virtues associated with relationships, both interpersonal and as articulated by policies and social norms”^58^ – such as the species’ contributions to individual and cultural identity within local community^59^. In this study, the story-telling about bears in the BAS area emphasized their inclusion as part of the history of the region, either through named individuals with whom people felt a connection^60^ or through long-lasting local legends. This symbolic value was also observed in central Italy, where residents with highly positive attitudes toward the Apennine brown bear expressed pride in having this species in the region^6^, suggesting that bears are valued as part of their collective identity. In their review on interventions to reduce human fear of large carnivores, Maria et al.^61^ suggest that increasing the exposure to large carnivores could affect people’s fear of the species, as it is an essential procedure for animal phobia treatment. Therefore, in areas where exposure to bears is now increasing or is expected to increase, promoting frequent positive interactions with bears could mediate people’s negative attitudes and behaviours^57^, helping establish a positive human-bear relationships^33,58^.

Our findings also suggest that in Spain, particularly in the areas where bears occur and the perception of benefits was the highest (BAS), there is a stronger acknowledgment and experience of the link between economic advantages associated with species conservation^9,24^. For example, mortality patterns of brown bears in the Cantabrian Mountains have changed with the recovery of the population^62^. While there are differences among regions^63^ and a significant amount of uncertainty about the causes of death in some areas, Balseiro et al.^62^ linked the decreasing levels of confirmed bear poaching with an increase in bear-related ecotourism in the region.

From another perspective, as it is common among public attitudes toward large carnivores, regions with more positive attitudes tend to correspond to those where perceived economic risk are lower^33,38,40^. Among European brown bear populations, those inhabiting Spain are among the least carnivorous^64^. Their low frequency of livestock consumption is mainly attributed to scavenging behaviour^65,66^. Thus, the low importance of wild ungulates and livestock in bear’s diet in those regions may help explain the low-risk perception. However, damages on beehives and orchards are frequent^23,67^. The implementation of prevention measures, spurred by local governments and several NGOs that operate in conflict-prone areas, may have helped reduce the risk of negative interactions with the species. Despite similar perceived risk levels, residents of bear-inhabited areas (BAS) expressed lower concern over bear proximity than those in non-bear areas (NBAS). This suggests that bear-related damages in their current distribution may be interpreted as an acceptable price to pay and outweighed by benefits associated with their presence.

### Apprehensive Portuguese response toward bear’s return

The overall neutral beliefs and perceptions of the Portuguese residents regarding the return of bears to Portugal contrast with the overwhelming support reported by Azevedo et al.^22^. Their study found that all surveyed actors viewed bears as non-dangerous, believed people were prepared to handle bear encounters and associated risks, and viewed the potential return of bears as a greater opportunity than a threat^22^. While our study also found generally positive attitudes toward the species among Portuguese residents, it revealed a more negative assessment of the bear’s presence, especially when considering the proximity to their properties. This evident pattern reflects a NIMBY effect regarding bears, similar to the findings by Lino et al.^51^ on the largest carnivore that currently occurs in Portugal, the Iberian wolf (*Canis lupus signatus*). In our survey, Portuguese residents were also divided in their perception of bear-related benefits and risks, as a considerable proportion of the sampled population acknowledged the risks associated with bear presence in the region, without considering its benefits. From the cultural perspective of Portuguese respondents, these perceptions are supported by the predominantly negative stories we recorded from the time when bears still roamed this landscape. The portrayal of bears as a dangerous and as a damage-causing species may contribute to less positive attitudes, as it is a clear expression of people’s fear of bears^52^, especially exacerbated in a context where there is no longer any direct familiarity with the species (e.g. references to bears attacking beehives to consume honey)^50^. The similarity in attitudes and perceptions of residents from both Portuguese study regions may stem from the belief that bears do not represent an immediate danger in either region. This aligns with the minimal damages that were reported during the confirmed bear incursion in 2019, as negative meaningful interactions are a known driver of intolerance^33,38^. However, contrary to what is observed in the Spanish regions where bears occur (BAS), Northern Portugal is characterized by a general lack of protection measures against predators^68^. Therefore, we argue that the resistance we found toward the re-establishment of the bear population in Portugal could worsen with new and more frequent incursions into Portuguese territory, as the perception of risks and, consequently, individual negative attitudes increase^69^. The discrepancy between our results and those reported by Azevedo et al.^22^ likely stems from differences in respondent socio-demographic characteristics. By surveying attendees at a workshop in Northen Portugal focused on the return of brown bears to the country, Azevedo et al.^22^ sampled individuals from different interest groups who were already engaged with the species conservation, and more incline to support the future presence of brown bear. While we acknowledge several limitations in our study, which we address in the following section, we consider that our results reflect an interpretation that more closely reflects the views of local communities.

### Limitations and future directions

Our findings highlighted the different perspectives and attitudes between Spanish and Portuguese respondents in light of the Cantabrian bear’s ongoing southern expansion. However, they should not be interpreted as a generalization of the beliefs, perceptions, and attitudes of the residents across the entire Iberian Peninsula. In this study, we were not able to fully analyse the social and spatial heterogeneity of people’s relationship with bears, as has been reported in other landscapes^6,7,9^ and within Spain^23,29,30^. First, historical cohabitation and exposure to bears are not homogeneous across the current bear distribution area in Spain. For example, Sage et al.^70^ in a study of spatial variability of grizzly bears’ acceptance by humans, found that the relationship between experiences with the species and acceptance differed depending on whether the analysis was conducted at the individual level or regional level. At a smaller spatial scale, certain attitudes can differ in response to environmental changes that may induce modifications of bears’ behavior^28,70^, such as bear recolonization. Second, due to this study’s focus on regional and national attitudinal differences, our sample was not designed to be representative of the populations’ socio-economic, education, gender, and age composition and, therefore, may be skewed. Particularly, individuals with a higher level of education were overrepresented, likely due to the online methods of distribution of the questionnaire.

Although we don’t have data on which distribution method (mailing list or social media) directed each respondent to the questionnaire, we assume both channels may have excluded individuals with a lower education level, who may have limited access to online tools. As such, our sample may be inherently biased toward individuals with more positive attitudes toward bears, as prior research^9,40,48^ indicates that knowledge is a strong predictor of favourable attitudes. Nevertheless, the differences found through our large-scale approach highlighted the need for distinct management strategies in Spain and Portugal. Implementing a common management model in a heterogeneous socio-cultural and attitudinal context could increase the risk of social conflicts^28^. Future approaches to the study of attitudes toward bears in these regions should aim to collect more robust and representative population samples to overcome these limitations. We expect that an analysis based on a more representative sample with higher spatial resolution would reveal a more complex pattern of attitudes across the Iberian Peninsula and allow a better tailored approach to minimize the conflict with bears.

In the current tipping point of the process of the brown bear’s expansion into Portugal, a more thorough understanding of how this may unfold is essential to delineate an effective management conservation plan for the species in the country. There is a lack of information on the suitable habitats for bear in Northern Portugal, considering both ecological and sociological components. To address this knowledge gap, we propose a multi-scale social-ecological approach to identify the possible complex expansion corridors’ scenarios. This account not only for the landscape composition dimension, but also include human perceptions, attitudes and behaviours. Our study highlights the need to identify the areas in Portugal where a positive relationship between humans and bears should be fostered and areas where more negative relationships may occur that require targeted and tailored interventions. Moreover, this pre-colonization context could be an opportunity to create an environment for participatory processes and shared responsibilities among academic institutions, government agencies, and local communities from the outset^69^ to forecast challenges and difficulties and identify solutions, in a proactive and not reactive approach. By doing so, the return of brown bears to Portugal can be positioned not as an imposition or a burden to local human populations, but as an added value to local wildlife biodiversity, to landscape functionality, and a tool to promote livelihoods.

## Conclusion

Our study highlights the complex and contrasting attitudes toward brown bears in the Iberian Peninsula. While Spanish respondents—particularly those in regions with established bear populations—held positive attitudes, Portuguese respondents demonstrated a more neutral stance, with greater apprehension about bears’ proximity to human settlements. The findings suggest that positive experiences and frequent encounters with bears play a crucial role in shaping public perceptions and fostering acceptance. In Spain, where bears are already integrated into local landscapes and economies, public support is reinforced by the perceived benefits of conservation and ecotourism. In contrast, Portugal, where bears have been absent for generations, exhibits more uncertainty and a NIMBY-like response to their potential return. As brown bears continue their expansion southward, these insights underscore the need for proactive conservation strategies in Portugal. The return of brown bears should not be viewed as a challenge but as an opportunity to enhance biodiversity, restore ecological balance, and promote sustainable livelihoods. By involving local communities, policymakers, and conservation organizations in early planning efforts, Portugal can ensure that bear recolonization is framed as a shared success rather than a source of division.

## Supplementary Information

Below is the link to the electronic supplementary material.

Supplementary Material 1

## Data Availability

The data presented in this study is available from the corresponding author upon reasonable request.
