# Supplementary material for: Positive perceptions of brown bears linked to long-term cohabitation in the Iberian Peninsula
Source: Sci Rep. 2025 Oct 2;15:34362. doi: 10.1038/s41598-025-16979-2 (PMC12491485; doi:10.1038/s41598-025-16979-2)
Supplement: Supplementary file 1 — Supplementary Material 1 [file 41598_2025_16979_MOESM1_ESM.docx]

**Supplementary materials**

for the manuscript “Positive perceptions of brown bears linked to long-term cohabitation in the Iberian Peninsula”

**Supplementary Data S1: Portuguese version of questionnaires**

O seguinte inquérito foi desenvolvido pelo Departamento de Biologia da Universidade de Aveiro no âmbito de um estudo sobre o Urso-pardo (doravante designado apenas como urso), cujo objetivo é conhecer o que as pessoas pensam em relação a esta espécie. Não existem respostas “certas” ou “erradas”, pois estamos interessados na sua opinião. Toda a informação recolhida é confidencial e manter-se-á anónima, mas é essencial para esta investigação. Completar o questionário demorará cerca de 10 minutos. A participação é voluntária e pode desistir a qualquer momento. Muito Obrigado!

Qualquer dúvida que tenha e/ou se quiser ter acesso posterior aos resultados desta investigação poderá contactar a equipa através do seguinte endereço eletrónico: Mariana Graça (gmariana@ua.pt) ou Nuno Negrões ([nunonegroes@ua.pt](mailto:nunonegroes@ua.pt))

| **1. Alguma vez viu um urso em cativeiro? (Jardins zoológicos, circos, etc.)** | |
| --- | --- |
| ◯ Nunca | ◯ Sim Se sim, quantas vezes (1, 2, 3...10...20)? |
| - 1. **Se respondeu “Sim” à pergunta anterior, como classifica essa experiência?**   (1 = Muito negativa; 2 = negativa; 3=Indiferente; 4 = Positiva; 5 = Muito positiva) | |
| **1** ◯ **2** ◯ **3** ◯ **4** ◯ **5** ◯ Porquê? | |

| **2. Alguma vez viu um urso em liberdade?** | |
| --- | --- |
| ◯ Nunca | ◯ Sim Se sim, quantas vezes (1, 2, 3...10...20...)? |
| - 1. **Se respondeu sim à pergunta anterior:** | |
| - - 1. **Onde foi a última vez?**     2. **Há quanto tempo?**     3. **Em que circunstâncias?** | |
| **2.1.4. Como classifica essa experiência?** (1 = Muito negativa; 2 = Negativa; 3 = Indiferente; 4 = Positiva; 5 = Muito positiva) | |
| **1** ◯ **2** ◯ **3** ◯ **4** ◯ **5** ◯ Porquê? | |

| **3- Assinale com um círculo a resposta que melhor descreve a sua opinião em relação a cada uma das seguintes frases:** | | | | | |
| --- | --- | --- | --- | --- | --- |
| **3.1. O urso desperta-me sentimentos** | Muito negativos | Negativos | Nem negativos nem positivos | Positivos | Muito positivos |
| **3.2. A existência de urso na região em que vivo seria** | Muito negativa | Negativa | Nem negativa nem positiva | Positiva | Muito positiva |
| **3.3- O urso é perigoso para o ser humano** | Discordo completamente | Discordo | Não concordo nem discordo | Concordo | Concordo completamente |
| **3.4- A presença de urso traria benefícios económicos para a região**  **onde vivo.** | Discordo completamente | Discordo | Não concordo nem discordo | Concordo | Concordo completamente |
| **3.5- O urso desempenha uma função ecológica importante, e é benéfico para a Natureza.** | Discordo completamente | Discordo | Não concordo nem discordo | Concordo | Concordo completamente |
| **3.6- A presença do urso na região aumenta o valor estético da paisagem**  **(enriquece o valor do património natural), quer eu o veja ou não.** | Discordo completamente | Discordo | Não concordo nem discordo | Concordo | Concordo completamente |
| **3.7- Parece-me bem que existam ursos na Europa.** | Discordo completamente | Discordo | Não concordo nem discordo | Concordo | Concordo completamente |

| **3.8- Parece-me bem que existam ursos em Portugal.** | Discordo completamente | Discordo | Não concordo nem discordo | Concordo | Concordo completamente |
| --- | --- | --- | --- | --- | --- |
| **3.9- Para mim é aceitável que haja ursos no concelho onde vivo.** | Discordo completamente | Discordo | Não concordo nem discordo | Concordo | Concordo completamente |
| **3.10 - A presença do urso é indicadora de um ambiente natural saudável.** | Discordo completamente | Discordo | Não concordo nem discordo | Concordo | Concordo completamente |
| **3.11- A presença do urso em Portugal apenas é admissível caso este não cause danos (ex. colmeias).** | Discordo completamente | Discordo | Não concordo nem discordo | Concordo | Concordo completamente |
| **3.12- O estado deverá cobrir os custos**  **de danos causados pelo urso, caso ocorram.** | Discordo completamente | Discordo | Não concordo nem discordo | Concordo | Concordo completamente |
| **3.13- O urso, tal como outros animais selvagens, tem o direito de viver em Portugal.** | Discordo completamente | Discordo | Não concordo nem discordo | Concordo | Concordo completamente |
| **3.14- Em áreas onde se registem danos**  **frequentes causados por urso, deverá ser permitido o abate de alguns destes.** | Discordo completamente | Discordo | Não concordo nem discordo | Concordo | Concordo completamente |
| **3.15- Os ursos deveriam viver apenas**  **em áreas protegidas ou reservas naturais.** | Discordo completamente | Discordo | Não concordo nem discordo | Concordo | Concordo completamente |
| **3.16- Para mim, é importante conservar as populações de urso para as gerações futuras.** | Discordo completamente | Discordo | Não concordo nem discordo | Concordo | Concordo completamente |
| **3.17- A presença do urso em Portugal seria algo muito positivo.** | Discordo completamente | Discordo | Não concordo nem discordo | Concordo | Concordo completamente |
| **3.18- Ver um urso na Natureza é uma**  **experiência entusiasmante.** | Discordo  completamente | Discordo | Não concordo  nem discordo | Concordo | Concordo  completamente |
| **3.19- O Homem tem o direito de usar**  **a Natureza de acordo com as suas necessidades.** | Discordo completamente | Discordo | Não concordo nem discordo | Concordo | Concordo completamente |
| **3.20- O Homem devia proteger a Natureza, porque os animais e plantas têm o direito de viver.** | Discordo completamente | Discordo | Não concordo nem discordo | Concordo | Concordo completamente |
| **3.21- Para mim proteger a Natureza não é importante.** | Discordo completamente | Discordo | Não concordo nem discordo | Concordo | Concordo completamente |

| **4- Em relação ao urso:** | |  |  | | |
| --- | --- | --- | --- | --- | --- |
| **4.1. Sabe se, no passado, existiu urso em Portugal?** | | | | | |
| ◯ Sim, e ainda existe atualmente | ◯ Sim, existiu no passado | | | ◯ Não sei |  |
| ◯ Não, nunca existiu urso em Portugal | |  |  | | |

| **4.2. O urso da Península Ibérica é aproximadamente do tamanho de:** | | | | | | | | | | | | |
| --- | --- | --- | --- | --- | --- | --- | --- | --- | --- | --- | --- | --- |
| ◯ Ovelha (± 100kg) | ◯ Porco (± 200kg) | | | | ◯ Vaca (± 500kg) ◯ Não sei | | | | |  |  |  |
| ◯ Outro: ______________________ | | |  | | | |  | | | | | |
|  | | |  | | | |  | | | | | |
| **4.3. Na Península Ibérica, dieta do urso é:** | | | | | | | | | | | | |
| ◯ Carnívora (outros animais) | | ◯ Herbívora (plantas e frutos) | | | | ◯ Omnívora (de tudo um pouco) | | | | | |  |
| ◯ Não sei | | |  | | | |  | | | | | |
|  | | |  | | | |  | | | | | |
| **4.4. Normalmente, o urso é um animal:** | | | | | | | | | | | | |
| ◯ Solitário (os machos e as fêmeas apenas se juntam para se reproduzirem) | | | | ◯ Que vive em grupos de 4 ou mais animais | | | | ◯ Não sei | | |  |  |
| ◯ Que vive em casais (macho e fêmeas formam  um casal estável) | | | | ◯ Outra: ____________________________ | | | | |  |  |  |  |

|  | |
| --- | --- |
|  |  |
|  | |

| **5- Em relação a si:** | |  | |  | | | | |
| --- | --- | --- | --- | --- | --- | --- | --- | --- |
| **5.1. Quanto tempo está em contacto com a Natureza (montanha, serra, bosque, etc.)?** | | | | | | | | |
| ◯ Todos os dias | ◯ Todos os meses | | | | ◯ Nunca | |  |  |
| ◯ Todas as semanas | | | ◯ Algumas vezes por ano | | | ◯ Outra: __________________________ | |  |

| **5.2. Onde costuma obter informação sobre o urso?** (Pode selecionar mais do que 1 opção) | **5.3. De 1 a 5, qual o nível de confiança que tem nessa fonte?**  (Classifique apenas as que selecionou)  (1 = Nenhuma confiança; 2 = Pouca confiança; 3 = Nem muita nem pouca (nível intermédio); 4 = Muita confiança; 5 = Total confiança) | | | | |
| --- | --- | --- | --- | --- | --- |
| ◯ Jardins zoológicos e Museus | **1** ◯ | **2** ◯ | **3** ◯ | **4** ◯ | **5** ◯ |
| ◯ Jornais | **1** ◯ | **2** ◯ | **3** ◯ | **4** ◯ | **5** ◯ |
| ◯ Televisão (telejornais, programas de natureza, …) | **1** ◯ | **2** ◯ | **3** ◯ | **4** ◯ | **5** ◯ |
| ◯ Redes Sociais (Facebook, Twitter, etc.) | **1** ◯ | **2** ◯ | **3** ◯ | **4** ◯ | **5** ◯ |
| ◯ Conversa com amigos ou familiares | **1** ◯ | **2** ◯ | **3** ◯ | **4** ◯ | **5** ◯ |
| ◯ Livros, revistas ou filmes | **1** ◯ | **2** ◯ | **3** ◯ | **4** ◯ | **5** ◯ |
| ◯ Organizações Não Governamentais (ONG) | **1** ◯ | **2** ◯ | **3** ◯ | **4** ◯ | **5** ◯ |
| ◯ Outra: | **1** ◯ | **2** ◯ | **3** ◯ | **4** ◯ | **5** ◯ |
| ◯ Não procuro/recebo informação sobre urso | | | | | |

| **6. Com que frequência obtém** | **informa** | **ção sobre o urso?** |  | |
| --- | --- | --- | --- | --- |
| ◯ Todos os dias  ◯ Uma vez por semana  ◯ Uma vez por mês | ◯  ◯  ◯ | Algumas vezes por ano Uma vez por ano  Nunca | ◯ Outra: | |
|  |  |  |  | |
| **7. A partir de que distância (km) da sua área de residência ficaria preocupado com a presença de urso?** | | | | ___________Km |

| **8-A presença de urso implica prejuízo financeiro para a região onde vivo.** | Discordo completamente | Discordo | Nem Discordo nem concordo | Concordo | Concordo completamente |
| --- | --- | --- | --- | --- | --- |

| **8.1- Se concorda com a frase anterior, que atividades seriam mais afetadas?** (Pode selecionar mais que 1 opção) | | | | | |
| --- | --- | --- | --- | --- | --- |
| ◯ Criação de gado | ◯ Agricultura | | ◯ Produção de mel ◯ Caça | |  |
| ◯ Outro: ______________________ | |  | |  | |

| **9. Realiza alguma destas atividades?** | | **9.1. Se sim, qual a relevância económica que essa atividade tem no seu rendimento económico?** | | | | | |
| --- | --- | --- | --- | --- | --- | --- | --- |
| **A. Produção de mel** | ◯ Sim | ◯ Menos de 10% | | ◯ 30 – 50% | | ◯ 70 – 90% | |
|  | ◯ Não | ◯ 10 – 30% | | ◯ 50 – 70% | | ◯ 90% – 100% | |
| **B. Agricultura** | ◯ Sim | ◯ Menos de 10% | | ◯ 30 – 50% | | ◯ 70 – 90% | |
|  | ◯ Não | ◯ 10 – 30% | | ◯ 50 – 70% | | ◯ 90% – 100% | |
| **C. Criação de gado (pode selecionar mais do que 1 opção)** | ◯ Caprino | ◯ Menos de 10%  ◯ 10 – 30% | | ◯ 30 – 50%  ◯ 50 – 70% | | ◯ 70 – 90%  ◯ 90% – 100% | |
|  | ◯ Cavalar | ◯ Menos de 10%  ◯ 10 – 30% | | ◯ 30 – 50%  ◯ 50 – 70% | | ◯ 70 – 90%  ◯ 90% – 100% | |
|  | ◯ Bovino | ◯ Menos de 10%  ◯ 10 – 30% | | ◯ 30 – 50%  ◯ 50 – 70% | | ◯ 70 – 90%  ◯ 90% – 100% | |
|  | ◯ Ovino | ◯ Menos de 10%  ◯ 10 – 30% | | ◯ 30 – 50%  ◯ 50 – 70% | | ◯ 70 – 90%  ◯ 90% – 100% | |
|  | ◯ Não |  | |  | |  | |
| **D. Caça** | ◯ Sim | | ◯ Menos de 10% | | ◯ 30 – 50% | | ◯ 70 – 90% |
|  | ◯ Não | | ◯ 10 – 30% | | ◯ 50 – 70% | | ◯ 90% – 100% |

| **10- Se faz criação de gado, alguma vez teve prejuízos causados pelo lobo ou pelo urso?** | | | |
| --- | --- | --- | --- |
| ◯ Não |  |  |  |
| ◯ Sim, pelo lobo | Por favor, descreva os detalhes sobre os mesmos __________________________________________________________  ____________________________________________________________________________________________________________________ | |  |
| ◯ Sim, pelo urso | Por favor, descreva os detalhes sobre os mesmos __________________________________________________________  ____________________________________________________________________________________________________________________ | |  |

| **11- Na sua infância existiam rumores, mitos ou histórias populares relacionadas com ursos? Se sim, quais?** |
| --- |

____________________________________________________________________________________________________________________________________________________________________________________________________________________________________________________________________________________________________________________________________________________________________________________________________________________________________________________________________________________________________________________________________________________________________________________________________________________________________________________________

| **12. Informação pessoal** | | | | |
| --- | --- | --- | --- | --- |
| **Género:** | ◯ Feminino | ◯ Masculino | **Idade:** | **Profissão:** |
| **Freguesia de residência:**   **Concelho de residência**:  **Código Postal**:  **Há quanto tempo vive no local?** | | | | |
| **Grau de escolaridade:** ◯ Sem Escolaridade ◯ Escola Primária ◯ 2º e/ou 3º ciclo | | | | |
| ◯ Secundário ◯ Ensino Superior ◯ Outra | | | | |

**Muito obrigado pela sua participação!**

**Supplementary Data S2: Spanish version of questionnaires**

Desde el Departamento de Biología de la Universidad de Aveiro estamos realizando un estudio sobre el oso pardo (de aquí en adelante oso) cuyo objetivo es conocer la opinión de la gente sobre esta especie. No hay respuestas "correctas" o "incorrectas", solo nos interesa su opinión. Toda la información recogida será confidencial y se analizará de manera agregada y se mantendrá el carácter anónimo de cada respuesta. Completar el cuestionario le llevará unos 10 minutos. La participación es voluntaria y puede retirarse en cualquier momento, pero esencial para esta investigación. ¡Muchas gracias!

Si tiene alguna pregunta y/o desea tener acceso a los resultados de esta investigación en una fecha posterior, puede ponerse en contacto con nuestro equipo de investigación a través de las siguientes direcciones de correo electrónico: Mariana Graça ([gmariana@ua.pt](mailto:gmariana@ua.pt)) o Nuno Negrões ([nunonegroes@ua.pt](mailto:nunonegroes@ua.pt))

| **1. ¿Has visto alguna vez un oso en cautividad?** (Zoológicos, circos, etc.) | |
| --- | --- |
| ◯ Nunca | ◯ Sí Si es así, ¿cuántas veces (1, 2, 3...10...20)? |
| - 1. **Si ha respondido afirmativamente a la pregunta anterior, ¿cómo califica esta experiencia?**   (1 = Muy negativa; 2 = Negativa; 3 = Indiferente; 4 = Positiva; 5 = Muy positiva) | |
| **1** ◯ **2** ◯ **3** ◯ **4** ◯ **5** ◯ ¿Por qué? | |

| **2. ¿Has visto alguna vez un oso en la naturaleza?** | |
| --- | --- |
| ◯ Nunca | ◯ Sí Si es así, ¿cuántas veces (1, 2, 3...10...20...)? |
| - 1. **Si ha respondido afirmativamente a la pregunta anterior:** | |
| - - 1. **¿Dónde fue la última vez?**     2. **¿Cuánto tiempo hace?**     3. **¿Qué circunstancias?** | |
| **2.1.4. ¿Cómo califica esta experiencia?** (1 = Muy negativa; 2 = Negativa; 3 = Indiferente; 4 = Positiva; 5 = Muy positiva) | |
| **1** ◯ **2** ◯ **3** ◯ **4** ◯ **5** ◯ ¿Por qué? | |

| **3- Marque con un círculo la respuesta que mejor describa su opinión sobre cada una de las siguientes frases:** | | | | | |
| --- | --- | --- | --- | --- | --- |
| **3.1. El oso me despierta sentimientos** | Muy negativos | Negativos | Ni negativos ni positivos | Positivos | Muy positivos |
| **3.2. La existencia del oso en la región donde vivo es:** | Muy negativa | Negativa | Ni negativos ni positiva | Positiva | Muy positiva |
| **3.3- El oso es peligroso para el ser**  **humano.** | Totalmente en  desacuerdo | Desacuerdo | Ni de acuerdo ni  en desacuerdo | De acuerdo | Totalmente De  acuerdo |
| **3.4- La presencia del oso aporta beneficios económicos a la región donde vivo.** | Totalmente en desacuerdo | Desacuerdo | Ni de acuerdo ni en desacuerdo | De acuerdo | Totalmente De acuerdo |
| **3.5- El oso cumple una importante**  **función ecológica y es beneficioso para la naturaleza.** | Totalmente en desacuerdo | Desacuerdo | Ni de acuerdo ni en desacuerdo | De acuerdo | Totalmente De acuerdo |
| **3.6- La presencia del oso en la región aumenta el valor estético del paisaje (enriquece el valor del patrimonio**  **natural).** | Totalmente en desacuerdo | Desacuerdo | Ni de acuerdo ni en desacuerdo | De acuerdo | Totalmente De acuerdo |
| **3.7- Me parece bien que haya osos en Europa.** | Totalmente en desacuerdo | Desacuerdo | Ni de acuerdo ni en desacuerdo | De acuerdo | Totalmente De acuerdo |
| **3.8- Me parece bien que haya osos en España.** | Totalmente en desacuerdo | Desacuerdo | Ni de acuerdo ni en desacuerdo | De acuerdo | Totalmente De acuerdo |
| **3.9- Para mí es aceptable que haya osos en la provincia donde vivo.** | Totalmente en desacuerdo | Desacuerdo | Ni de acuerdo ni en desacuerdo | De acuerdo | Totalmente De acuerdo |
| **3.10 - La presencia del oso es un indicador de un entorno natural**  **saludable.** | Totalmente en desacuerdo | Desacuerdo | Ni de acuerdo ni en desacuerdo | De acuerdo | Totalmente De acuerdo |
| **3.11- La presencia del oso en España sólo es admisible si no causa daños (ej. colmenas).** | Totalmente en desacuerdo | Desacuerdo | Ni de acuerdo ni en desacuerdo | De acuerdo | Totalmente De acuerdo |
| **3.12- El estado debe cubrir los costes de los daños causados por el oso, cuando los hay.** | Totalmente en desacuerdo | Desacuerdo | Ni de acuerdo ni en desacuerdo | De acuerdo | Totalmente De acuerdo |
| **3.13- El oso, como otros animales salvajes, tiene derecho a vivir en**  **España.** | Totalmente en desacuerdo | Desacuerdo | Ni de acuerdo ni en desacuerdo | De acuerdo | Totalmente De acuerdo |
| **3.14- En las zonas donde el oso causa daños con frecuencia, se debería permitir matar algunos de estos animales para controlarlos.** | Totalmente en desacuerdo | Desacuerdo | Ni de acuerdo ni en desacuerdo | De acuerdo | Totalmente De acuerdo |
| **3.15- Los osos deberían vivir sólo en zonas protegidas o reservas naturales.** | Totalmente en desacuerdo | Desacuerdo | Ni de acuerdo ni en desacuerdo | De acuerdo | Totalmente De acuerdo |
| **3.16- Para mí, es importante preservar las poblaciones de osos para las**  **generaciones futuras.** | Totalmente en desacuerdo | Desacuerdo | Ni de acuerdo ni en desacuerdo | De acuerdo | Totalmente De acuerdo |
| **3.17- La presencia del oso en España es algo muy positivo.** | Totalmente en desacuerdo | Desacuerdo | Ni de acuerdo ni en desacuerdo | De acuerdo | Totalmente De acuerdo |
| **3.18- Ver un oso en la naturaleza es una experiencia emocionante.** | Totalmente en desacuerdo | Desacuerdo | Ni de acuerdo ni en desacuerdo | De acuerdo | Totalmente De acuerdo |
| **3.19- El hombre tiene derecho a utilizar la naturaleza según sus necesidades.** | Totalmente en desacuerdo | Desacuerdo | Ni de acuerdo ni en desacuerdo | De acuerdo | Totalmente De acuerdo |
| **3.20- El hombre debe proteger la naturaleza, porque los animales y las**  **plantas tienen derecho a vivir.** | Totalmente en desacuerdo | Desacuerdo | Ni de acuerdo ni en desacuerdo | De acuerdo | Totalmente De acuerdo |
| **3.21- Proteger la naturaleza no es importante para mi.** | Totalmente en desacuerdo | Desacuerdo | Ni de acuerdo ni en desacuerdo | De acuerdo | Totalmente De acuerdo |

| **4- Em relação ao urso:** |  |  |
| --- | --- | --- |

| **4.1. El oso de la Península Ibérica tiene aproximadamente el tamaño de:** | | | | | | | | | | | |
| --- | --- | --- | --- | --- | --- | --- | --- | --- | --- | --- | --- |
| ◯ Oveja (± 100kg) | | ◯ Cerdo (± 200kg) | | ◯ Vaca (± 500kg) | | | | ◯ Otro: | ◯ No lo sé | | |
| **4.2. En la Península Ibérica, la dieta del oso es:** | | | | | | | | | | | |
| ◯ Carnívora (otros animales) | | | ◯ Herbívora (plantas y frutos) | | | ◯ Omnívora (un poco de todo) | | | | ◯ No lo sé |  |
| **4.3. Normalmente, el oso es un animal:** | | | | | | | | | | | |
| ◯ | Solitario (los machos y las hembras sólo se juntan para reproducirse) | | | | ◯ | | Que vive en grupos de 4 o más animales | | ◯ No lo sé | | |
| ◯ | Que vive en pareja (el macho y la hembra forman una pareja estable) | | | | ◯ | | Otro: | |  | | |

| **5- Algunas preguntas sobre usted:** | | | | | | | | |
| --- | --- | --- | --- | --- | --- | --- | --- | --- |
|  | | | | | | | | |
| **5.1. ¿Cuánto tiempo está en contacto con la naturaleza (montaña, monte, bosque, etc.)?** | | | | | | | | |
| ◯ Todos los días |  | ◯ Todos los meses |  | ◯ Nunca | | | | |
| ◯ Todas las semanas |  | ◯ Algunas veces al año | | ◯ Otro: | | | | |
| **5.2. ¿Dónde suele obtener información sobre el oso? (Puede seleccionar más de una opción)** | | | **5.3. Del 1 al 5, ¿qué nivel de confianza tiene en esa fuente? (Califique sólo los que haya seleccionado)**  (1 = Ninguna confianza; 2 = Poca confianza; 3 = Ni mucha ni poca (nivel intermedio); 4 = Mucha confianza; 5 = Total  confianza) | | | | | |
| ◯ Zoológicos y museos | | |  | **1** ◯ | **2** ◯ | **3** ◯ | **4** ◯ | **5** ◯ |
| ◯ Periódicos | | |  | **1** ◯ | **2** ◯ | **3** ◯ | **4** ◯ | **5** ◯ |
| ◯ Televisión (telediarios, programas de naturaleza, ...) | | |  | **1** ◯ | **2** ◯ | **3** ◯ | **4** ◯ | **5** ◯ |
| ◯ Redes sociales (Facebook, Twitter, etc.) | | |  | **1** ◯ | **2** ◯ | **3** ◯ | **4** ◯ | **5** ◯ |
| ◯ De amigos o familiares | | |  | **1** ◯ | **2** ◯ | **3** ◯ | **4** ◯ | **5** ◯ |
| ◯ Libros, revistas o películas | | |  | **1** ◯ | **2** ◯ | **3** ◯ | **4** ◯ | **5** ◯ |
| ◯ Organizaciones no gubernamentales (ONG) | | |  | **1** ◯ | **2** ◯ | **3** ◯ | **4** ◯ | **5** ◯ |
| ◯ Otros: | | |  | **1** ◯ | **2** ◯ | **3** ◯ | **4** ◯ | **5** ◯ |
| ◯ No busco/recibo información sobre el oso | | | | | | | | |
|  | | | | | | | | |
| **6. ¿Con qué frecuencia obtiene información sobre el oso?** | | | | | | | | |
| ◯ Todos los días | ◯ Algunas veces al año | | ◯ Otra: | | | | | |
| ◯ Una vez a la semana | ◯ Una vez al año | |  |  | | | | |
| ◯ Una vez al mes | ◯ Nunca | |  |  | | | | |

**7. ¿A qué distancia (en kilómetros) de su zona de residencia le preocuparía la presencia del oso?**  Km

| **8- La presencia del oso causas pérdidas económicas en la región en que vivo.** | Totalmente en desacuerdo | Desacuerdo | Ni de acuerdo ni en desacuerdo | De acuerdo | Totalmente De acuerdo |
| --- | --- | --- | --- | --- | --- |

| **8.1- Si está de acuerdo con la frase anterior, ¿qué actividades se verían más afectadas? (Puede seleccionar más de una opción)** | | | | |  |
| --- | --- | --- | --- | --- | --- |
| ◯ Ganadería | ◯ Agricultura | ◯ Colmenas | ◯ Caza | ◯ Otras: ________________________________ | |

| **9. ¿Está involucrado en alguna de estas**  **actividades?** | | **9.1. En caso afirmativo, ¿qué relevancia económica tiene esta actividad en**  **sus ingresos económicos?** | | |
| --- | --- | --- | --- | --- |
| **A. Producción de miel** | ◯ Sí | ◯ Menos de 10% | ◯ 30 – 50% | ◯ 70 – 90% |
|  | ◯ No | ◯ 10 – 30% | ◯ 50 – 70% | ◯ 90% – 100% |
| **B. Agricultura** | ◯ Sí | ◯ Menos de 10% | ◯ 30 – 50% | ◯ 70 – 90% |
|  | ◯ No | ◯ 10 – 30% | ◯ 50 – 70% | ◯ 90% – 100% |
|  | ◯ Caprino | ◯ Menos de 10% | ◯ 30 – 50% | ◯ 70 – 90% |
|  |  | ◯ 10 – 30% | ◯ 50 – 70% | ◯ 90% – 100% |
| **C. Ganadería**  **(puede seleccionar más de una opción)** | ◯ Caballo | ◯ Menos de 10%  ◯ 10 – 30% | ◯ 30 – 50%  ◯ 50 – 70% | ◯ 70 – 90%  ◯ 90% – 100% |
|  | ◯ Vacuno | ◯ Menos de 10%  ◯ 10 – 30% | ◯ 30 – 50%  ◯ 50 – 70% | ◯ 70 – 90%  ◯ 90% – 100% |
|  | ◯ Ovino | ◯ Menos de 10% | ◯ 30 – 50% | ◯ 70 – 90% |
|  |  | ◯ 10 – 30% | ◯ 50 – 70% | ◯ 90% – 100% |

|  | ◯ No | |
| --- | --- | --- |
| **D. Caza** | ◯ Sí  ◯ No | ◯ Menos de 10% ◯ 30 – 50% ◯ 70 – 90%  ◯ 10 – 30% ◯ 50 – 70% ◯ 90% – 100% |

| **10- Si cría ganado, ¿ha tenido alguna vez pérdidas causadas por el lobo u oso?** | | | |
| --- | --- | --- | --- |
| ◯ No |  |  |  |
| ◯ Si de lobo | Por favor proporcione detalles de las perdidas__________________________________________________________  ____________________________________________________________________________________________________________________ | |  |
| ◯ Si de oso | por favor proporcione detalles de las perdidas___________________________________________________________  ____________________________________________________________________________________________________________________ | |  |

| **11- ¿En su infancia había leyendas, mitos o historias populares relacionadas con los osos? Si es así, ¿cuáles?** |
| --- |

_______________________________________________________________________________________________________________________________________________________________________________________________________________________________________________________________________________________________________________________________________________________________________________________________________________________________________________________________________________________________________________________________________

**¡Muchas gracias por su participación!**

| **12. Información personal** | | | | |
| --- | --- | --- | --- | --- |
| **Género:** | ◯ Femenino | ◯ Masculino | **Edad:** | **Profesión:** |
| **Municipio de residencia:**   **Provincia de residencia:**   **Código Postal:**   **¿Cuánto tiempo llevas viviendo allí? _____________** | | | | |
| **Nivel de estudios finalizado:** ◯ Ninguno ◯ EGB, Primaria ◯ FP, BUP, secundaria | | | | |
| ◯ Universidad ◯ Otros | | | | |

**Supplementary Table S1**

Table S1: Chi-Squared test results for association between demographic variables and attitudes/perceptions toward brown bears and their presence

| Demographic variables | **Attitudes toward bears** | | | **Perceptions** | | **Attitudes toward bear presence** |
| --- | --- | --- | --- | --- | --- | --- |
|  | Affective Component | Cognitive Component | | Benefit | Risk |  |
| Gender* | p < 0,05; V = 0,18 | p = 0,066 | - | p < 0,01; V = 0,24 | p < 0,01; V = 0,21 | p < 0,01; V= 0,19 |
| Age** | p < 0,05; V = 0,18 | p =0,20 | - | p < 0,01; V = 0,27 | p < 0,01; V= 0,24 | p < 0,05; V = 0,19 |

*Female or Male

**18-39 years old, 40-59 years old, ≥ 60 years old
